# Genome-Wide Gene Expression Disturbance by Single A1/C1 Chromosome Substitution in *Brassica rapa* Restituted From Natural *B. napus*

**DOI:** 10.3389/fpls.2018.00377

**Published:** 2018-03-20

**Authors:** Bin Zhu, Yang Xiang, Pan Zeng, Bowei Cai, Xiaolong Huang, Xianhong Ge, Qingbei Weng, Zaiyun Li

**Affiliations:** ^1^School of Life Sciences, Guizhou Normal University, Guiyang, China; ^2^National Key Laboratory of Crop Genetic Improvement, National Center of Oil Crop Improvement (Wuhan), College of Plant Science and Technology, Huazhong Agricultural University, Wuhan, China; ^3^Guizhou Rapeseed Institute, Guizhou Academy of Agricultural Sciences, Guiyang, China; ^4^Key Laboratory of Plant Physiology and Development Regulation, School of Life Sciences, Guizhou Normal University, Guiyang, China

**Keywords:** aneuploidy, chromosome substitution, *Brassica napus*, *B. rapa*, transcriptome

## Abstract

Alien chromosome substitution (CS) lines are treated as vital germplasms for breeding and genetic mapping. Previously, a whole set of nine *Brassica rapa-oleracea* monosonic alien addition lines (MAALs, C1-C9) was established in the background of natural *B. napus* genotype “Oro,” after the restituted *B. rapa* (RBR) for Oro was realized. Herein, a monosomic substitution line with one alien C1 chromosome (Cs1) in the RBR complement was selected in the progenies of MAAL C1 and RBR, by the PCR amplification of specific gene markers and fluorescence *in situ* hybridization. Cs1 exhibited the whole plant morphology similar to RBR except for the defective stamens without fertile pollen grains, but it produced some seeds and progeny plants carrying the C1 chromosome at high rate besides those without the alien chromosome after pollinated by RBR. The viability of the substitution and its progeny for the RBR diploid further elucidated the functional compensation between the chromosome pairs with high homoeology. To reveal the impact of such aneuploidy on genome-wide gene expression, the transcriptomes of MAAL C1, Cs1 and euploid RBR were analyzed. Compared to RBR, Cs1 had sharply reduced gene expression level across chromosome A1, demonstrating the loss of one copy of A1 chromosome. Both additional chromosome C1 in MAAL and substitutional chromosome C1 in Cs1 caused not only *cis*-effect but also prevalent *trans*-effect differentially expressed genes. A dominant gene dosage effects prevailed among low expressed genes across chromosome A1 in Cs1, and moreover, dosage effects for some genes potentially contributed to the phenotype deviations. Our results provided novel insights into the transcriptomic perturbation and gene dosage effects on phenotype in CS related to one naturally evolved allopolyploid.

## Introduction

Increasing narrow genetic diversity due to selection pressure excessively focusing on quality and yield in the crop’s breeding pools has been a big challenge for breeders to enhance current levels of resistance to biotic and abiotic stresses (Chang and de Jong, 2005; Gupta et al., 2016). Therefore, plant breeders and geneticists are greatly interested in enhancing genetic variants of crops via interspecific and somatic hybridization to transfer desirable traits from wild species (Quiros et al., 1987; Du et al., 2009; Heneen et al., 2012; Chen et al., 2014; Kang et al., 2014; Zhu et al., 2016). As intermediate crossing products of interspecific hybridization, alien chromosome addition lines, CS lines and other aneuploidy lines not only offer the ideal opportunity to produce introgression lines, but provide a unique avenue to check heterologous gene expression and interaction between recipient genome and donor chromosome in plants (Barthes and Ricroch, 2001; Zhu et al., 2016). However, few studies referring to gene expression in these aneuploidy lines have been carried out so far.

Aneuploidy generally manifests itself as impaired fitness, abnormal development and even lethal to organism due to the burden of additional or insufficient gene products (Siegel and Amon, 2012). The typical example in human is Down’s syndrome, which is arisen from extra copy of entire or partial chromosome 21, resulting in a cognitive impairment, muscle hypotonia, as well as dysmorphic features (Letourneau et al., 2014). The vast majority of human cancers also display various levels of aneuploidy. Despite the fact that plants have better tolerance to the aneuploidy than animals, all aneuploid plants grow more poorly than euploid plants (Singh et al., 1996; Henry et al., 2010; Zhu et al., 2015). Recently, gene expression patterns in aneuploidy organisms were gradually noted. With the high-throughput technologies, studies of global gene expression in aneuploidy plants and animals (Huettel et al., 2008; Zhang et al., 2010; Mäder et al., 2011; Letourneau et al., 2014) demonstrated that the *trans*-acting effects across remainder genome were found to be quite prevalent, rather than the *cis*-acting effects along altered chromosomes.

Monosomic CS lines generally derived from progenies of alien addition lines have been believed to enhance homoeologous chromosomes to pair and synapse (Chang and de Jong, 2005), making it a powerful genetic resource for transferring desirable traits and analyzing the contribution of chromosome segments to phenotypic variations (Saha et al., 2006; Gupta et al., 2016). However, because of the molecular mechanism of inhibition of homoeologous recombination existing in allopolyploid (Feldman and Levy, 2012; Buggs et al., 2014), as well as much more risk of death in CS lines, it is much more difficult to generate CS lines than alien additions. Impressively, stocks of addition lines and viable substitution lines have been developed in a variety of crop plants. A plenty of chromosomal substitution lines of common wheat carrying alien chromosomes have been established to transfer traits like diseases tolerance and superior environmental adaption into varieties for several decades (Wells et al., 1982; Du et al., 2015; Pang et al., 2014; Zhu et al., 2017). Rice carrying the chromosomal segment of common wild rice has been essential to enhance the tolerance to chilling (Wang et al., 2017). Some chromosomal segment substitution lines have also been developed and performed in isolation of alleles of rice target QTLs (Wan et al., 2006; Yang et al., 2015). The contribution of alien substituted chromosomes with tolerance to root-knot nematode has also been documented in upland cotton (*Gossypium hirsutum* L) carrying Pima cotton (*G. barbadense* L) chromosomes (Ulloa et al., 2016). Recently, C-genome CS lines in derived *B. juncea* with the increased genetic diversity and hybrid performance had been identified by cytogenetic and molecular methods (Gupta et al., 2016).

The diploids *B. rapa* L. (2n = 2x = 20, A^r^A^r^) and *B. oleracea* L. (2n = 2x = 18, C^o^C^o^) arising from a common hypothetical hexaploid ancestor (Cheng et al., 2013) showed closed genome relatedness and parented the economically valuable allotetraploid *B. napus* (2n = 38, A^n^A^n^C^n^C^n^) (Nagaharu, 1935; Chalhoub et al., 2014). Compared to *B. rapa, B. oleracea* shows stronger resistance to biotic and abiotic stresses. Therefore, interspecific hybridization between *B. rapa* and *B. oleracea* was performed in many studies, attempting to transfer desirable genes from *B. oleracea* to *B. rapa* (Quiros et al., 1987; McGrath and Quiros, 1990; Prakash et al., 2009; Geleta et al., 2012). Intriguingly, in our previous study, the restituted B. rapa (RBR, 2n = 20, AnAn) ancestor was generated from natural *B. napus* through inducing the preferential elimination of C-subgenome chromosomes in intertribal crosses (Tu et al., 2010; Zhu et al., 2016), and subsequently the whole set of monosonic alien addition lines (MAALs) was established to *in situ* dissect C-subgenome by adding each of its nine chromosomes to the extracted A-subgenome (Zhu et al., 2016).

In this study, a substituted plant (2n = 20 = 19A+1C1) was picked out among these progenies of MAAL C1 and RBR, and RNA-seq analysis indicated that one chromosome of A1 was replaced in the CS line. Compared to euploid RBR, genome-wide gene expression revealed both *cis*-effect and prevalent *trans*-effect DEGs in MAAL C1 and CS line. Moreover, a dominant gene dosage effects prevailing among low expressed genes across chromosome A1 in Cs1 was also observed. These findings provided new insights into heterologous gene expression and interplay between recipient genome and donor chromosome.

## Materials and Methods

### Plant Materials

A complete set of *B. rapa-oleracea* MAALs with each of nine C-subgenome chromosomes added to the restituted RBR Oro was established in the background of natural *B. napus* genbotype “Oro” in our previous study (Zhu et al., 2016). A plant with 2n = 20 from the progenies of MAAL C1 after pollinated by RBR Oro was demonstrated to harbor one chromosome C1 by the PCR amplification of C1 chromosome specific gene markers and FISH with the C genome specific probe. This plant showed severe deficiency on stamen but were female fertile and produced some seeds after pollinated by RBR Oro. These plants were grown in the green house in a control condition in Huazhong Agriculture University.

### Morphology and Cytology Analysis

Morphological characteristics of the substitution line, MAAL C1 and RBR Oro were documented and compared in pairs. To determine the chromosome numbers of backcrossing progenies, ovaries from young flower buds were collected and treated with 2 mM 8-hydroxyquinoline for 3 h at room temperature, then fixed in Carnoy solution (3:1 ethanol: glacial acetic acid, v/v) and stored at -20° for use.

### PCR Amplification of C1 Chromosome Specific Gene Markers

Total DNA was extracted from young leaves according to the CTAB method. To confirm whether the backcrossing progenies harbored the chromosome C1, C1 chromosome specific gene markers (Zhu et al., 2016) were performed to amplify. The PCR reactions system was in a volume of 10 μl containing 1 × *Taq* buffer, 2 mM MgCl_2_, 2.5 mM dNTPs, 5 μM forward and reverse primer, 0.35 U *Taq* DNA polymerase and 50 ng genomic DNA. DNA fragments were amplified using an initial 5-min denaturation at 94° followed by 30 cycles (94° for 45 s, 53° -57° for 30 s, 72° for 45 s), and a final 10-min elongation step at 72°. Finally, PCR products were separated by 1% agarose gels.

### Probe Labeling and FISH Analysis

The plasmid DNA of BAC BoB014O06 specific for *Brassica* C-genome (provided by Susan J. Armstrong, University of Birmingham, Birmingham, United Kingdom) was labeled with biotin-11-dCTP by random priming using the BioPrime DNA Labeling System kit (Invitrogen, Life Technologies) according to the manufacturer’s protocol (Invitrogen, Life Technologies). Slide preparations were carried out mainly according to the methods of Zhong et al. (1996), and the BAC-FISH analyses was performed according to the procedure of Cui et al. (2012), with slight modification to reduce the washing time to 8 min in 0.1× saline sodium citrate (SSC) with 20% deionized formamide at 40°.

Images from FISH were captured using a computer-assisted fluorescence microscope with a CCD camera (Axio Scope A1, Zeiss, Germany). Photographs were manipulated by the software of Adobe Photoshop 7.0 (Adobe Systems, Inc.) to adjust the contrast and brightness and change the background into black.

### RNA Extraction and Preparation of cDNA Libraries

For RBR Oro, MAAL C1 and the substitution line, three biological replicates were used to prepare a total of nine cDNA libraries. Briefly, the third leaves from three plants per sample were pooled equally and ground in the liquid nitrogen. Total RNA was extracted using commercial RNA kit (Tiangen, China) according to the manufacturer’s protocol. The quality and quantity of isolated RNA were assessed by Qubit (Invitrogen, Life Technologies), and then the RNA Integrity Number (RIN) value was assessed by Agilent Technologies 2100 Bioanalyzer (Agilent). 1.5 μg RNA per biological replicate was used to prepare the c-DNA library following the TruSeq RNA Sample Prep v2 protocol when the RNA Integrity Number value was higher than 8. Subsequently, the nine libraries were sequenced on Illumina HiSeqTM 3000 platform (Illumina, United States) to generate 150 bp paired-end reads.

### Differentially Expressed Genes (DEGs) Analysis

Trimmomatic software version 0.33 (Bolger et al., 2014) was used to remove adapters and low quality reads to generate clean reads with following parameters: LEADING: 3, TRAILING: 3, SLIDINGWINDOW: 4:15, MINLEN: 36, LEADING: 3, TRAILING: 3, SLIDINGWINDOW: 4:15, MINLEN: 36, TOPHRED: 33. Then, these clean reads were aligned to the *B. napus* reference genome with 101040 predicted genes (Chalhoub et al., 2014) using Hisat software (version 0.1.6) with a strict mismatch tolerance according to the follow criterion: specifying -L, 0, -0.15 (Kim et al., 2015). FPKM (Fragments Per Kilobase per Million mapped reads) value was calculated using Cufflinks (version 2.2.1) with default parameters and performed to calculate gene expressions (Trapnell et al., 2012). To assess the differences in gene expression level among substitution line, MAAL C1 and RBR Oro in pairs, the package of DEseq2 with the cutoff of twofold change in gene expression and false discover rate with *q* < 0.05 (Trapnell et al., 2013) was performed to pick out DEGs. Eventually, the GO annotations and enrichments for DEGs were determined by Blast2GO (Conesa et al., 2005) and the R package of GOseq (FDR, *p* < 0.05), respectively.

### Data Statistics

The significance of data statistics, including Wilcoxon signed-rank test, Pearson Correlation Coefficient as well as chi-square test (χ^2^-test) was determined by the data analysis function of R project. The RNA-seq data for the present study is available at Gene Expression Ombinus (GEO) with the accession number GSE108122.

## Results

### Morphology of Substitution Line

**FIGURE 1:**
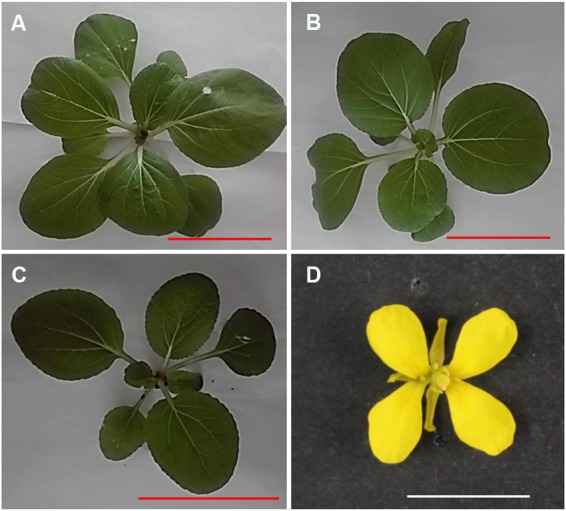
Morphology of aneuploidy RBR Oro, MAAL C1 and Cs1 at seedling stage and flower of Cs1. **(A–C)** Young seedlings for RBR Oro, MAAL C1 and Cs1, bar: 5 cm. **(D)** The Cs1 plant shows abnormal flower with defective stamen, bar: 1 cm.

From 52 backcrossing plants between MAAL C1 and RBR as pollen donor, a substituted plant (1.92%) (2n = 19A + 1C) was initially picked out via chromosome specific gene markers and counting chromosome number, while that which chromosome was substituted was still undetermined. Compared to MAAL C1 and euploid RBR Oro, the substitution plant showed the similar oval, glabrous, convex and dark green leaves to RBR Oro but the smallest plant size (**Figures 1A–C**) and the most delayed flowering time, about seven and 10 days later than MAAL C1 and RBR Oro, respectively. It also showed severe defective stamens (**Figure 1D**) with no fertile pollen grains but produced 73 seeds (1.46 seeds per pod) after pollination by RBR Oro. By PCR amplifications of chromosome C1 specific gene markers, 32 out of surviving 72 plants (44%) carried the chromosome C1 which was transmitted via female gamete, was slight higher than that in MAAL C1 (0.31, 15/48), but not so significant (χ^2^-test, *P* = 0.23). This indicated that female aneuploidy gamete (9A + 1C) was also highly viable in the competition with the euploidy gamete (10A). No fragmental chromosome C1 added was detected in the offspring.

### Identity of the Substituted Chromosome

**FIGURE 2:**
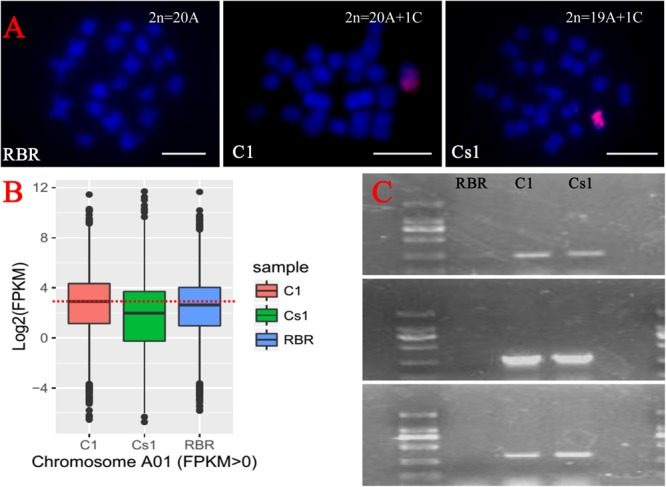
Detection of the identity of the substitution line from the backcrossing between MAAL C1 and RBR Oro by FISH, PCR amplification and RNA-seq analysis. **(A)** *Blue color* is for DAPI staining and *red signal* is from the labeled BAC BoB014O06 probe. The FISH analysis shows a chromosome configuration from mitotic cell with 19A chromosomes and 1C chromosome in substitution line. Bar: 10 μm. **(B)** Box plot of Log_2_(FPKM) of the total expressed genes (FPKM > 0) along on chromosome A1 indicates one A1 chromosome missing. **(C)** PCR amplifications of C1 chromosome specific gene markers confirms that the C1 chromosome retains in the substitution line. The sizes of marker ladder from top to end are 2000, 1000, 750, 500, 250, and 100 bp, respectively.

To detect whether the substitution line harbored one structural intact C1 chromosome, five C1 chromosome specific gene markers located on its two arms (Zhu et al., 2016) were amplified and all the amplification products of these markers were present (**Figure 2C**). Then by FISH analyses with BAC BoB014O06 as probe which was specific to C-subgenome chromosome in *Brassica* (Cui et al., 2012; Zhu et al., 2015), the chromosomal constitution of 19 A-subgenome chromosomes and one C-subgenome chromosome was demonstrated (**Figure 2A**).

To further determine which chromosome of A genome was replaced in the substitution line, we performed RNA-seq to check the gene expression reduction for one particular chromosome. Because of high homoeology between A1 and C1 chromosomes (Xiong et al., 2011), the chromosome A1 was most likely the one being substituted. As expected, compared to MAAL C1 and euploid RBR Oro, this substitution line showed sharply reduced gene expression level along chromosome A1, as indicated by their box plots of expressed genes (FPKM > 0, FPKM, Fragments per Kilobase of transcript per Million mapped reads) (**Figure 2B**).

### Differentially Expressed Genes in Three Paired Comparisons

To draw a comprehensive picture of the perturbations of gene expression in the substitution line, we performed its RNA-seq data and compared to MAAL C1 and RBR Oro. The substitution line, MAAL C1 and RBR Oro were abbreviated as Cs1, C1 and RBR, respectively. After the adapters and the low-quality reads were removed by Trimmomatic software version 0.33, 7.46–18.43 million clean 150-bp end pair reads per sample were generated. Then these clean data were mapped to the *B. napus* genome with 101040 predicted genes (Chalhoub et al., 2014). Finally, 74.14–77.30% of clean reads per sample, including 15.30–19.00% multiple mapped reads were aligned to the reference genome (**Table 1**).

**Table 1:** Summary of clean reads per sample mapped to reference genome of *B. napus* ‘Darmor-bzh.’

Sample	Clean reads	Unique mapped (%)	Multiple mapped (%)	Total mapped (%)
RBR_1	9492588	61.50	15.80	77.30
RBR_2	9217222	59.16	16.25	75.41
RBR_3	7458828	60.73	15.87	76.60
C1_1	9066801	58.97	15.30	74.27
C1_2	10939481	58.82	15.59	74.41
C1_3	10637980	59.21	15.30	74.51
Cs1_1	14850829	56.75	17.39	74.14
Cs1_2	18433830	56.04	18.34	74.38
Cs1_3	14929375	55.88	19.00	74.88

**FIGURE 3:**
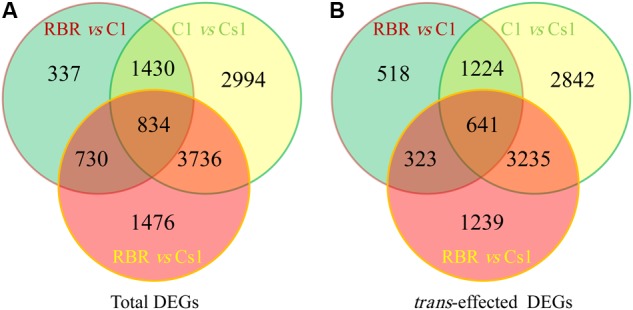
Total and *trans*-effected differentially expressed genes (DEGs) from three comparisons are shown in Venn diagram (http://bioinformatics.psb.ugent.be/webtools/Venn/). **(A)** Venn analysis for total DEGs. **(B)** Venn analysis for *trans*-effected DEGs.

After that, the values of FPKM per sample were assessed to determine the gene expression profiling among Cs1, C1 and RBR. And the DEseq2 package software was performed to determine the DEGs. Compared to euploid RBR, 3331 DEGs were identified in C1, including 1561 (46.86%) up-regulated genes and significantly higher down-regulated genes (1770, 53.14%) (χ^2^-test, *P* = 2.93e-4). Remarkably, more than double DEGs (6776), including 3433 (50.66%) up-regulated genes and comparable down-regulated genes (3343, 49.34%) (χ^2^-test, *P* = 0.27), were detected in the comparison of RBR vs. Cs1, probably attributing to the simultaneous chromosome loss and substitution in Cs1. 8994 DEGs, including 4259 (47.35%) down-regulated genes and a significant bias to up-regulated genes (4735, 52.65%) (χ^2^-test, *P* = 5.19e-5) were also found in the comparison C1 vs. Cs1. The results of Venn diagram supported by the public webtools (Bioinformatics & Evolutionary Genomics^1^) showed that 1564 DEGs were identified both in RBR vs. C1 and RBR vs. Cs1, almost half of total DEGs in RBR vs. C1 (46.95%) and quarter of DEGs in RBR vs. Cs1 (**Figure 3A**). Detailed information of DEGs was summarized in **Table 2**.

**Table 2:** Proportion of up- and down-regulated differentially expressed genes (DEGs) in three comparisons.

	RBR *vs*. C1		C1 *vs*. Cs1		RBR *vs*. Cs1	
	DEGs	Ratio (%)	DEGs	Ratio (%)	DEGs	Ratio (%)
Up-regulated	1561	46.86	4735^∗∗^	52.65	3433	50.66
Down-regulated	1770^∗∗^	53.14	4259	47.35	3343	49.34
Total	3331		8994		6776	

*^∗∗^Groups extremely significantly different detected by chip-seq, *p* < 0.01.*

### *Cis*- and *Trans*-Effects for Transcripts in Aneuploids C1 and Cs1

Although MAAL C1 had an extra chromosome C1, it showed an extremely significant bias to down-regulated genes in comparison with RBR. This suggested not only a strong systemic effect for the unbalanced chromosome complement (*cis*-effect), but also a wide range of *trans*-effects for transcripts on the other disomic chromosomes (*trans*-effects). This result was in accordance with recent studies of gene expression patterns in aneuploidy (Huettel et al., 2008; Letourneau et al., 2014; Zhu et al., 2015).

To assess the *trans*-effects of unbalanced chromosomes on gene expression, we picked out DEGs along disomic chromosomes in the comparisons of RBR vs. C1, RBR vs. Cs1 and C1 vs. Cs1. For RBR vs. C1, 2706 (81.24%) DEGs were on the 20 disomic chromosomes, including 950 (37.36%) up-regulated genes and 1756 (62.64%) down-regulated genes, with an extremely significant bias to the latter (χ^2^-test, *P* = 2.20e-16). From the comparison RBR vs. Cs1, after ruling out the DEGs on extra chromosome C1 and deficient chromosome A01, 5438 (80.25%) DEGs were detected, including 2586 (47.21%) up-regulated genes and 2852 down-regulated genes (52.79%), with a significant bias to down-regulation (χ^2^-test, *P* = 3.10e-4). The comparison of C1 vs. Cs1 gave 8122 (90.30%) DEGs belong to trans-effects (**Table 3**).

**Table 3:** Summary of *trans*-effected DEGs in three comparisons.

	RBR vs. C1		C1 vs. Cs1		RBR vs. Cs1	
	DEGs	Ratio (%)	DEGs	Ratio (%)	DEGs	Ratio (%)
Up-regulated	950	37.36	4480^∗∗^	53.41	2586	47.21
Down-regulated	1756^∗∗^	62.64	3642	46.59	2852^∗∗^	52.79
Total	2706		8122		5438	

*^∗∗^Groups extremely significantly different detected by chip-seq, *p* < 0.01.*

Whether these *trans*-effect DEGs presented random contributions on all disomic chromosomes in the substitution line? Thus, we assessed the proportion of genome-wide expressed genes and DEGs of the remainder disomic chromosomes in two comparisons. *Trans*-effects did not appeared equally across all chromosomes in the comparisons of RBR vs. C1 and RBR vs. Cs1. Intriguingly, chromosome A03 were always most susceptible in both comparisons, but chromosome A02 and chromosome A04 were most stable (**Supplementary Table S1**, χ^2^-test, *P* < 0.001), hinting that aneuploidy might impact gene expression distinctly on some chromosomes.

**FIGURE 4:**
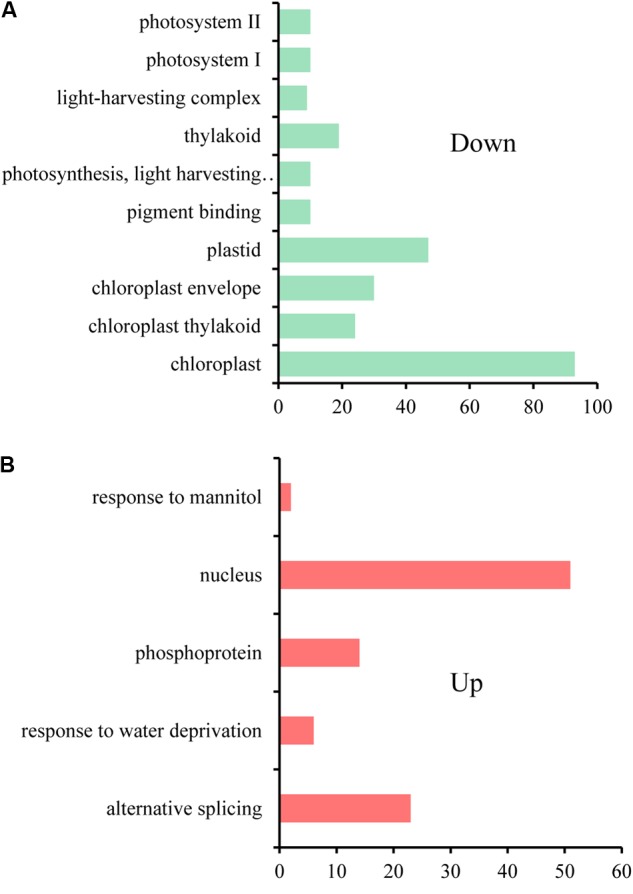
The gene ontology analysis for common *trans*-effected DEGs in the comparisons of RBR vs. C1 and RBR vs. Cs1. **(A)** The gene annotation terms for down regulated genes. **(B)** The gene annotation terms for up regulated genes.

Only a minor degree of *cis*-effect genes was observed, with 625 (18.76%) DEGs along on chromosome C1 in the comparison of RBR vs. C1 and 704 (10.39%) DEGs along on chromosome C1, as well as 634 (9.36%) DEGs across chromosome A01 in the comparison of RBR vs. Cs1. Interestingly, despite the reduced gene dosage, 5.19% (194) of genes along chromosome A01 in Cs1 had significantly higher expression levels than in the euploid RBR, probably attributing to the genome-wide dosage-compensation. These up-regulated genes, which were largely rather well-distributed along chromosome A01, appeared to have no conspicuous common features. To gain deeper insights into common patterns of the aneuploidy effect on gene expression, we performed Venn diagram to pick out the common-effected DEGs and specific DEGs. The identified 964 common *trans*-effect DEGs occupied 35.62% *trans*-effect DEGs in RBR vs. C1 and 17.72% *trans*-effect DEGs in RBR vs. Cs1. The comparisons of RBR vs. C1, RBR vs. Cs1 and C1 vs. Cs1 resulted in 518, 1239 and 2842 DEGs, respectively (**Figure 3B**). Taking into consideration of altered patterns of gene expressions, 273 down-regulated and 124 up-regulated trans-effected DEGs were detected both in RBR vs. C1 and RBR vs. Cs1. The gene ontology analysis revealed that these common DEGs, especially the reduced expressed genes (**Figure 4A**) mainly involved in “photosystem II” (Benjamini–Hochberg FDR, *P* = 9.9e-11), “thylakoid” (*P* = 1.1e-11), “photosynthesis, light harvesting in photosystem I”(*P* = 6.5e-13) as well as “pigment binding” (*P* = 2.8e-13) and in particular those involved in “plastid” (*P* = 4.5e-14) and “chloroplast” (*P* = 3.8e-19), indicating that aneuploidy had severe impairment on photosynthesis. Intriguingly, up-regulated common DEGs were demonstrated to be associated with the annotation cluster “response to mannitol” (*P* = 3.5e-2), “nucleus” (*P* = 1.6e-2), “phosphoprotein” (*P* = 6.6e-3) and “response to water deprivation” (*P* = 5.2e-3) and particular in “alternative splicing” (*P* = 1.8e-3), implying that the aneuploidy defect also triggered epigenetic alteration on genome- wide gene expression which needed further study (**Figure 4B**).

### Expression of One-Dose Genes in Cs1 Line

Gene dosage effects have been demonstrated to widely contribute to the phenotype alterations (Birchler and Veitia, 2007; Huettel et al., 2008). The substitution line provided an excellent chance to determine the overall patterns of one-dose genes response on chromosome A01. According to Malone et al.’s (2012) work, using *t*-test (*p* < 0.05), a null hypothesis, in which the expression of one-dose genes was twofold reduction in RBR compared to the Cs1 values for each gene, was tested to classify the expression of one-dose genes into three groups: anti-compensated, non-compensated and compensated. Briefly, genes for which the null hypothesis was not rejected, were stratified into non-compensated. The genes for which the null hypothesis was rejected and expression was lower than the expected two-fold reduction were treated as anti-compensated. And genes for which the null hypothesis was rejected and the expression was higher than the expectations were classified as compensation levels.

Our data showed that non-compensation (1558, 41.66%) class and non-expressed (1298, 34.71%) genes across chromosome A1 in Cs1 accounted for the most genes, however, 693 (18.53%) genes belonged to anti-compensated group. And only a minor degree of compensated genes (191, 5.10%) were observed. Additionally, we wondered if there was a clear correlation between gene expression levels and compensation state. Herein, based on the gene expression in RBR, we classified genes along chromosome A01 into three categories, according to low (0.1 < RPKM < 10, 1372 genes), medium (10 < RPKM < 100, 751 genes), or high (RPKM > 100, 96 genes) levels. Notably, non-compensation was more prevalent in low expression genes (918, 66.91%) and medium (403, 53.66%) groups (χ^2^-test, *P* < 0.05), while compensated genes, including anti-compensated genes were dominant in high group (49, 51.04%), but not significant (χ^2^-test, *P* = 0.84). However, compared to compensated genes, anti-compensated genes were prevalent among all expression levels (**Table 4**).

**Table 4:** The relationship between gene expression level and gene expression compensation.

Gene groups	Non-compensated	Ratio (%)	Compensated	Ratio (%)	Anti-compensated	Ratio (%)	Total
Low (0.1 < RPKM < 10)	918^∗∗^	66.91	96	7	358	26.09	1372
Medium (10 < RPKM < 100)	403^∗^	53.66	80	10.65	268	35.69	751
High (RPKM > 100)	47	48.96	15	15.63	34	35.42	96

*^∗∗^Groups extremely significantly different detected by chip-seq, *p* < 0.01. *^∗^*Groups significantly different detected by chip-seq, *p* < 0.05.*

### GO Annotation of Total and *Trans*-Effect DEGs

To further study the biological pathways that probably contributed to morphology alteration in MAAL C1 and Cs1, the DEGs were annotated in GO database. Due to insufficient gene function annotations in *B. napus*, the DEGs were first annotated by sequence alignment with homologous gene in TAIR 10 (The Arabidopsis Information Resources) (*E*-value cutoff of 1e-5). Totally, 2903 (87.15%) of 3331 DEGs (1574 down-regulated and 1329 up-regulated genes) in RBR vs. C1 and 5896 (87.01%) of 6776 DEGs (2919 down-regulated and 2977 up-regulated genes) in RBR vs. Cs1 were respectively aligned to their putative orthologs in *Arabidopsis thaliana*. Then these genes groups were assigned to Gene Ontology (GO) category with the three major terms, cellular component, molecular function and biological process via the open access functional annotation tool DAVID^2^ according to Benjamini modified FDR value (*P* < 0.05).

**FIGURE 5:**
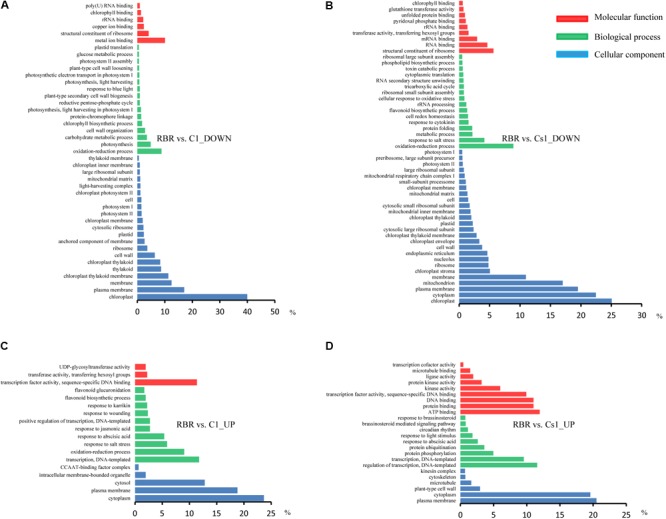
GO analysis of trans-effected DEGs in two comparisons of RBR vs. C1 and RBR vs. Cs1 annotated by three categories: cellular component (*blue*), molecular functions (*red*) and biological process (*green*). **(A,B)** Go annotation for down-regulated genes in both comparisons. **(C,D)** The reduced GO terms were clustered in up-regulated genes in both comparisons.

For the down-regulated genes, the GO terms related to chloroplast and photosystem were significantly clustered for both comparisons, indicating aneuploidy generally resulted in defective photosynthesis. The gene ontology analysis also revealed that the reduced expressed genes in C1 and Cs1 involved in “rRNA binding,” “structural constituent of ribosome” and “large ribosomal subunit” (**Figures 5A,B**), implying a reduction in protein synthesis in aneuploidy, consistent with the results of previous gene expression studies in other aneuploidy organisms (Torres et al., 2008; Stingele et al., 2012; Sheltzer et al., 2012). Particularly, sharply reduced GO terms were clustered in up-regulated genes in both RBR vs. C1 and RBR vs. Cs1, despite the fact that up-regulated DEGs and down-regulated genes were comparable in RBR vs. C1 and RBR vs. Cs1 (**Figures 5C,D**), indicating that the responses of highly expressed genes to aneuploidy impact were more random. The terms with “nucleus” and “positive regulation of transcription, DNA-templated” were revealed in both comparisons, which implied positive DNA synthesis and potentially supported the hypothesis that the structure of genome was undergoing a period of instability in aneuploidy (Huettel et al., 2008). Another finding was that the up-regulated genes in RBR vs. Cs1 were rich in the terms “response to brassinosteroid” and “brassinosteroid mediated signaling pathway” (**Figure 5D**), which provided some clues that brassinosteroid probably contributed to the morphology of substitution line with defective stamen.

To gain insights into the impact of aneuploidy on remainder gene expressions, the GO analysis was conducted to independently annotate the *trans*-effected DEGs. 2376 of 2706 *trans*-effected DEGs in RBR vs. C1 and 4742 of 5438 *trans*-effected DEGs in RBR vs. Cs1 were respectively aligned to their homologous partners in *A. thaliana*. Similar with the total DEGs annotation, the GO analysis revealed that reduced expressed genes were mainly involved in photosynthesis and protein synthesis related terms (**Supplementary Figures S1A,B**) in both comparisons. And up-regulated *trans*-effected genes were dominantly associated with “positive regulation of transcription, DNA-templated.” Additionally, up-regulated *trans*-DEGs involved in stress response were consistently observed (**Supplementary Figures S1C,D**). The terms “response to brassinosteroid” and “brassinosteroid mediated signaling pathway” associated with the up-regulated trans-effected DEGs were also observed in RBR vs. Cs1, indicating *trans*-effect mainly contributed to this cluster.

## Discussion

In present study, a monosomic CS line of *B. rapa* involving two highly homoeologous chromosomes A1 and C1 was established and identified through the methods of cytology and RNA-seq. The uniqueness of the substitution was that it contained the 19 A-subgenome chromosomes and 1 C-subgenome chromosome from the natural *B. napus* genotype, which provided the particular opportunity to study the genome interplay and aneuploidy effect on phenotype and gene expression in one allopolyploid with a long history. Homoeologous pairings and exchanges between A and C sub-genome due to their close relatedness were extensively revealed in synthetic and natural *B. napus* (Szadkowski et al., 2010; Cui et al., 2012; Chalhoub et al., 2014; Zhang et al., 2015). Particularly, homoeologous chromosome replacement and compensation frequently occurred between chromosome sets with extensive homoeology (A1/C1, A2/C2) to maintain chromosome dosage balance (Xiong et al., 2011). Our MAAL C1 (A^n^A^n^+ 1C^n^) not only showed the highest rates of allosyndetic trivalents, but also the higher transmission rate of the C1 chromosome by both male and female gametes (Zhu et al., 2016), which enhanced the possibility for establishment of CS line. But no such CS line has been reported in the development of addition lines from the crosses between natural *B. rapa* (A^r^A^r^) and *B. oleracea* (C^o^C^o^) (Geleta et al., 2012; Heneen et al., 2012). Potentially, closer relationship between A^n^ and C^n^ subgenomes in natural *B. napus* than that between A^r^ and C^o^ subgenomes from two independently evolved diploids enhanced the compensation between A^n^ and C^n^ subgenomes chromosomes and the viability of the CS plants. Alternatively, those CS progenies failed to be identified, owing to the previous technical limitations.

The CS lines and alien additions provided a unique avenue to investigate the heterologous gene expression and interplay between recipient genome and donor chromosome. It is very interesting to detect ectopic expression of alien genes, their dependence of genes along other chromosomes of the donor species, as well as the effect of different genetic backgrounds of the recipient genome (Chang and de Jong, 2005). However, few researches referring to gene expression and molecular level have been carried out probably due to shortage of genomic information of donor species. Recently, several genes related to steroidal saponin pathway were revealed in *Allium fistulosum* – *Allium cepa* monosomic addition lines through high-throughput RNA-seq (Abdelrahman et al., 2017), showing a feasible way to detect gene expression patterns of alien genes and gene expression interplay between heterologous genes. Because of a perturbation of stoichiometric relationships between gene products resulting in dosage imbalances (Birchler and Veitia, 2007), adding or subtracting of single chromosome usually produce stronger alteration of phenotypes than whole-genome change in organism. Herein, transcriptomic changes in both MAAL C1 and Cs1 via RNA-seq not only determined the origin of altered chromosome (*cis*-effect), but showed more extensively impact of altered chromosome on global gene expression (*trans*-effect), in line with the previous results of genome-wide perturbations of gene expressions from other aneuploids in *Arabidopsis*, fruit fly, human and *B. napus* (Huettel et al., 2008; Malone et al., 2012; Letourneau et al., 2014; Zhu et al., 2015). Besides, compared to MAAL C1, more than double DEGs occurred in Cs1, probably for a more complex karyotype resulted in more severe dosage imbalance (one C1 chromosome substituting one A1 chromosome). We also noticed that the impact of aneuploidy on gene expression was uneven across all chromosomes in both comparisons, implying that *trans*-effect of altered chromosome on remainder genome was under some special molecular mechanisms. Moreover, significant richment of the common upregulated genes in both comparisons in the term “alternative splicing” indicated that epigenetic alterations of wide-genome genes were triggered by unbalanced genome.

Gene dosage has been demonstrated to widely contribute to the phenotype alteration, while gene expression change was not consistent with the alteration of gene copy number (Birchler and Veitia, 2007; Huettel et al., 2008). The mechanisms responsible for the phenomenon were unrevealed. The engineered fruit flies (*Drosophila melanogaster*) where gene dose was reduced from two to one have been used as a model to detect the molecular mechanism under gene expression compensation (Malone et al., 2012). However, the detection of gene dosage change only strictly limited to few genes because chromosome or chromosome segmental alteration for autosome chromosome was generally fatal in human and animals. It is long recognized that plants have better tolerance to the aneuploidy than animals (Siegel and Amon, 2012). The whole chromosome aneuploidy even complete sets of trisomics, monosomics and nullisomics have been developed for some plants (Sears, 1954; Huettel et al., 2008; Zhu et al., 2015, 2016), which provided excellent chances to determine the overall pattern of dose responses of genes on copy altered chromosomes. In *A. thaliana*, a minor degree of dosage compensation (3%) was observed in chromosome 5 trisomics using array comparative genome hybridization (Huettel et al., 2008). Similarly, compensated genes along on altered chromosome A1 (23.63%) in Cs1 were also in the minority, implying a similar mechanism responsible in aneuploid. Interestingly, we also demonstrated that the compensated genes were prevalent in high expression genes. Gene expression compensation and anti-compensation were believed to be mediated by feedback or buffering in expression networks (Malone et al., 2012), probably attributing to epigenetic silencing, altered transcription factor availability, or some other masked mechanisms (Huettel et al., 2008).

Previously, the study for gene expression of a nullisomic_C2 in same *B. napus* genotype also observed not only clear *cis*-effects but prevalent *trans*-effects (Zhu et al., 2015). Compared to gene expression patterns change in nullisomic_C2 (91.77%), both C1 (81.23%) and Cs1 (80.25%) gave rise to relative low *trans*-effects, indicating that aneuploidy with insufficient gene products resulted in more severe impairment on gene expression than aneuploidy with additional and substituted gene products. Besides, several dysregulated domains which were clustered by either up-regulated or down-regulated expressed genes along same chromosomes were observed in the comparison of RBR vs. Cs1. This result was in line with the observation in monozygotic twins discordant for trisomy 21 which showed similar but much more dysregulated domains across all chromosomes (Letourneau et al., 2014). Once we believed the dysregulated domains of gene expression was a common feature for aneuploidy (Zhu et al., 2015), however, compared to euploid RBR, no such domains were clearly observed in Cs1 and C1, indicating that the dysregulation domains were likely specific to particular aneuploidy organisms.

## Conclusion

The generation of one chromosome substituted *B. rapa* plant (2n = 19A+1C) made it feasible to detect heterologous gene expression and interplay between recipient A subgenome and extra C1 chromosome. Subsequently, genome-wide genes expression not only determined the identity of replaced chromosome in A subgenome, but demonstrated existence of *cis*-effect DEGs along altered chromosome(s) and prevalent *trans*-effect DEGs across disomic chromosomes in both MAAL C1 and Cs1, which provided a unprecedent insights into gene expression interplay in heterogeneous aneuploidy (comprising of two genomes). The undergoing studies, particular in epigenetic studies, might reveal the molecular mechanisms responsible for the gene expression in such aneuploidy.

## Author Contributions

BZ designed this study and wrote the manuscript. YX analyzed the RNA-seq data. PZ, BC, and XH phenotyped and identified parental RBR “Oro” and C1 and the substitution line. XG provided the analysis of FISH for all materials. QW and ZL edited the manuscript, and all contributors have commented and approved the final manuscript.

## Conflict of Interest Statement

The authors declare that the research was conducted in the absence of any commercial or financial relationships that could be construed as a potential conflict of interest.

## Footnotes

1 http://bioinformatics.psb.ugent.be/webtools/Venn/

2 https://david.ncifcrf.gov/summary.jsp

## Supplementary Material

FIGURE S1 GO analysis of total DEGs in two comparisons of RBR vs. C1 and RBR vs. Cs1. **(A,B)** The terms clustered for down-regulated genes in both comparisons. **(C,D)** The terms clustered for up-regulated genes.

TABLE S1 *Trans*-effects across all disomic chromosomes in the comparison of RBR vs. C1 and RBR vs. Cs1.

## Abbreviations

CS: chromosome substitution
DEGs: differentially expressed genes
FISH: fluorescence *in situ* hybridization
HE: homoeologous exchanges
MAAL: monosomic alien addition line
RBR: restituted *B. rapa.*
