# Supplementary figures and images for: Genome-Wide Gene Expression Disturbance by Single A1/C1 Chromosome Substitution in Brassica rapa Restituted From Natural B. napus
Source: Front Plant Sci. 2018 Mar 20;9:377. doi: 10.3389/fpls.2018.00377 (PMC5870043; doi:10.3389/fpls.2018.00377)

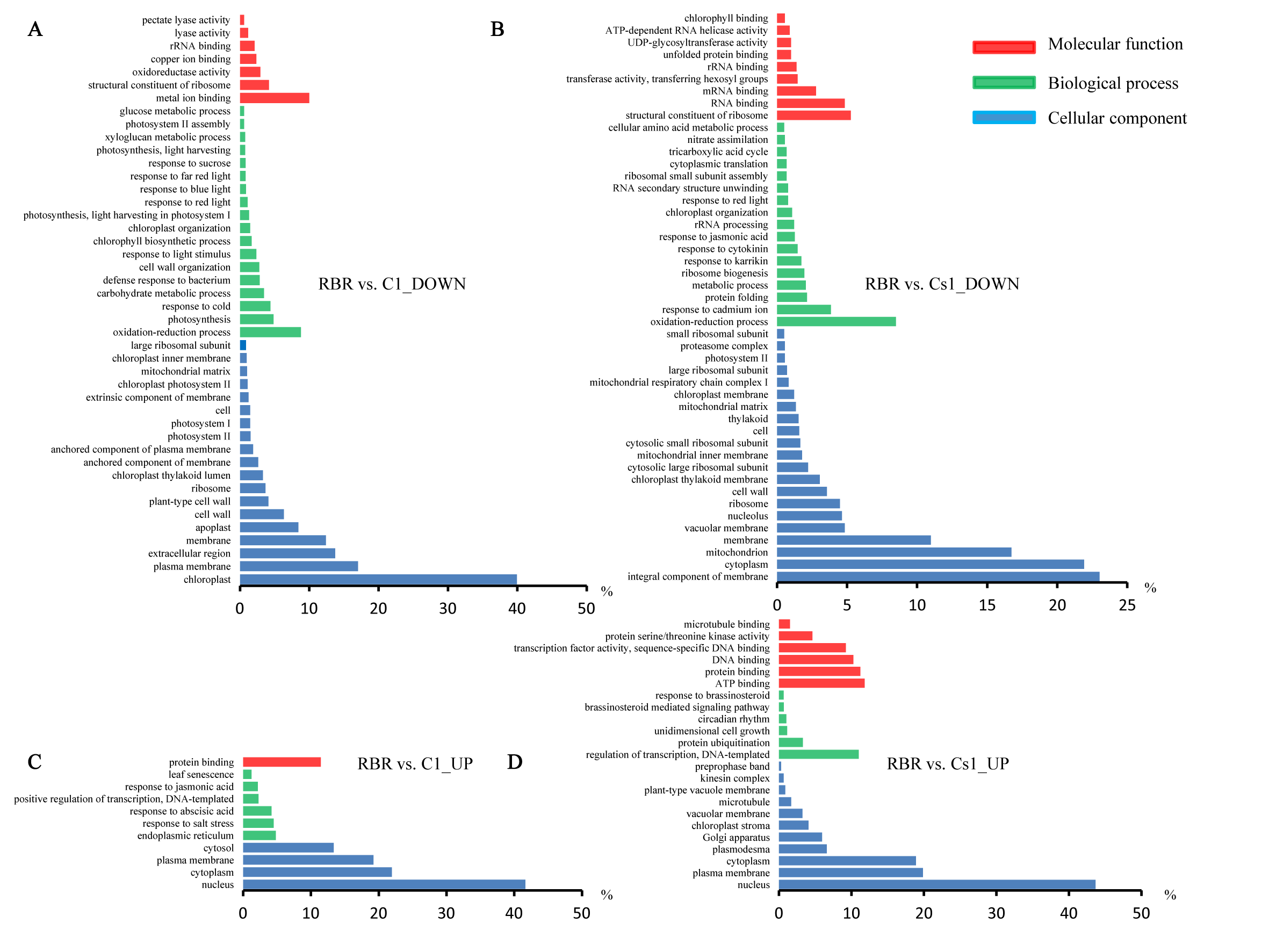

Supplement: FIGURE S1 — GO analysis of total DEGs in two comparisons of RBR vs. C1 and RBR vs. Cs1. (A,B) The terms clustered for down-regulated genes in both comparisons. (C,D) The terms clustered for up-regulated genes. [file Image_1.TIF]
